# Peripheral Artery Disease in the Colombian Orinoquía: Epidemiologic Profile From a Resource-Limited Hemodynamics Unit

**DOI:** 10.7759/cureus.101674

**Published:** 2026-01-16

**Authors:** Oscar F Vargas, Juliana Salcedo-Mesa, Valentina Lugo-Mesa, Laura Álvarez, Daniel Felipe Mesa Salcedo

**Affiliations:** 1 Interventional Radiology, Hospital Departamental de Villavicencio, Villavicencio, COL

**Keywords:** cross-sectional study, endovascular procedures, intermittent claudication, interventional, ischemia, peripheral arterial disease, prevalence, radiology

## Abstract

Introduction:Peripheral artery disease (PAD) mainly compromises the lower limbs and may progress to chronic limb-threatening ischemia (CLTI); in this context, endovascular management has become a cornerstone of revascularization strategies. This study aimed to describe the epidemiologic, clinical, and procedural characteristics of patients with PAD treated in a Hemodynamics Unit in 2023 at a resource-limited hospital in Colombia.

Methods: We conducted a retrospective observational descriptive study at a regional referral hospital in the Orinoquía region of Colombia. All adults who underwent an endovascular procedure for PAD between January 1 and December 31, 2023, were included. Patient-level variables were analyzed for 115 patients, and procedure-level variables for 152 PAD-related interventions. Demographic, clinical, and procedural data were extracted from electronic medical records. Descriptive statistics were performed, and exploratory chi-square tests were used to evaluate associations between clinical classifications, comorbidities, procedural outcomes, and limb amputations. Data analysis was performed using RStudio, Version 2024.12.1 + 563 (Posit PBC, Boston, MA, USA).

Results: During the study period, 1,984 procedures were performed, of which 152 (7.7%) corresponded to PAD-related endovascular interventions in 115 patients. The majority of patients were male, comprising 68 (59.1%). The most common comorbidities were dyslipidemia in 93 (80.9%), hypertension in 84 (73.0%), and diabetes mellitus in 71 (61.7%) patients. Preoperative pharmacological treatment most frequently included antihypertensive therapy in 75 (65.2%) and high-intensity statins in 51 (44.25%) patients. CLTI was the main indication for intervention, documented in 89 (58.55%) cases. Complications of the procedures were found in 11 (7.24%) cases, and reinterventions were required in 23 (15.13%) cases. Among the observed statistical associations, clinically relevant findings included the association between insulin-dependent diabetes mellitus and limb amputation (χ² = 6.2805, p = 0.043), as well as the association between the Global Limb Anatomic Staging System (GLASS) and limb amputation (χ² = 30.078, p < 0.001). Another statistically significant association was observed between the Wound, Ischemia, foot Infection (WIfI) classification and procedural complications (χ² = 87.889, p < 0.001).

Conclusions: PAD interventions were associated with low complication and reintervention rates, supporting the safety of endovascular management for this type of disease. Clinical classification systems showed significant associations with limb amputation, highlighting the importance of baseline disease severity determination. Additionally, this study provides a relevant epidemiological profile of PAD management within this regional context.

## Introduction

Peripheral artery disease (PAD) is a manifestation of systemic atherosclerosis that mainly compromises the lower extremities [1]. Its clinical presentation often includes intermittent claudication, characterized by lower-limb pain with straining due to insufficient distal arterial blood flow, a manifestation of atherosclerotic obstruction [1]. PAD may affect the aortoiliac, femoropopliteal, or infrapopliteal arterial segments, or it may involve multiple segments simultaneously [2]. As the disease progresses, the patient's risk of developing chronic limb-threatening ischemia (CLTI) increases: CLTI is a severe condition associated with high morbidity and major adverse limb events [1].

PAD affects approximately 230 million individuals worldwide, and its incidence is rising due to the increase of major cardiovascular risk factors in the population, such as type 2 diabetes mellitus and hypertension [3]. PAD is one of the leading causes of morbidity in the world, along with myocardial infarction and stroke; this highlights its importance not only in the clinical setting but also in the public health sector [4].

Optimal treatment of PAD requires a detailed diagnostic evaluation, assessment of the degree of arterial involvement, and individualized therapeutic strategies [5]. In the last decade, endovascular management has become the cornerstone of revascularization for infrapopliteal arteries, offering a less invasive alternative to open surgical approaches [4]. Endovascular management is associated with decreased morbidity, shorter hospital stays, and lower rates of limb amputation, according to recent literature [5]. Interventional radiology offers procedural safety and precision through imaging and minimally invasive techniques.

Despite advances in this field, there is a lack of epidemiological and clinical data in low- and middle-income settings. Characterization of risk factors and clinical profiles in a population-specific setting is relevant, especially for developing appropriate clinical guidelines, optimizing resources, reducing complications, and developing public health strategies in Latin America.

This study is therefore aimed at describing the epidemiologic, clinical, and procedural characteristics of patients with PAD treated in a Hemodynamics Unit in the Orinoquía region of Colombia during 2023.

## Materials and methods

A retrospective observational study with a cross-sectional baseline analysis was designed to estimate the prevalence of PAD-related endovascular procedures performed in the Interventional Radiology Unit, describe the demographic, clinical, angiographic, and procedural characteristics of patients undergoing endovascular treatment for PAD, and explore unadjusted associations between comorbidities, clinical classification systems, and limb amputation outcomes. The study was conducted at the Hospital Departamental de Villavicencio, a regional referral center in the Orinoquia region of Colombia. The study period extended from January 1 to December 31, 2023.

All patients aged 18 years or older who underwent an endovascular procedure for PAD during the study period were eligible for inclusion. Patients were excluded if they underwent open surgical treatment instead of an endovascular procedure, had a documented allergy to iodinated contrast, were pregnant, or had incomplete medical records. To ensure patient confidentiality, all electronic medical records were fully de-identified prior to data extraction. A non-probabilistic consecutive sampling strategy was used, incorporating every eligible case treated during the study period.

Data were retrospectively retrieved from electronic medical records using the DINÁMICA software system. The variables collected included demographic characteristics such as age and sex; comorbidities, including diabetes mellitus, hypertension, and hypothyroidism; clinical and angiographic classifications, such as the Rutherford and Global Limb Anatomic Staging System (GLASS) when available; procedural characteristics, such as type of endovascular intervention; and pre- and post-operative pharmacological management. Mortality events were verified through the Colombian National Registry at the end of the study period. Duplicate records were identified and removed based on patients' national identification numbers.

Data analysis was performed using RStudio Version 2024.12.1 + 563 (Posit PBC, Boston, MA, USA). The primary outcome was the proportion of PAD-related procedures, which was calculated in relation to the total number of procedures performed in the Interventional Radiology Unit, including reinterventions. Secondary outcomes included procedural intervention results and classifications, as well as preprocedural demographic and clinical characteristics. Patient-level variables (demographics, comorbidities, and baseline clinical classifications when documented) were analyzed using the patient as the unit of analysis (N=115). Procedure-level variables (treated limb, target segment, devices, complications, reinterventions, and postoperative medications) were analyzed using the procedure as the unit of analysis (N=152). Descriptive statistics were used to summarize demographic, clinical, and procedural characteristics. Categorical variables were reported as frequencies and percentages, while continuous variables were reported as means or medians, depending on the distribution. Inferential analyses included chi-square tests to examine associations among comorbidities, clinical classification systems, and limb amputation; all tests were reported with their corresponding chi-square values and p-values. All inferential analyses were exploratory and unadjusted; no multivariable models were performed due to sample size limitations.

The study protocol was reviewed and approved by the Institutional Ethics Committee of Hospital Departamental de Villavicencio and complied with national and institutional guidelines for research involving human subjects.​

## Results

**Figure 1:**
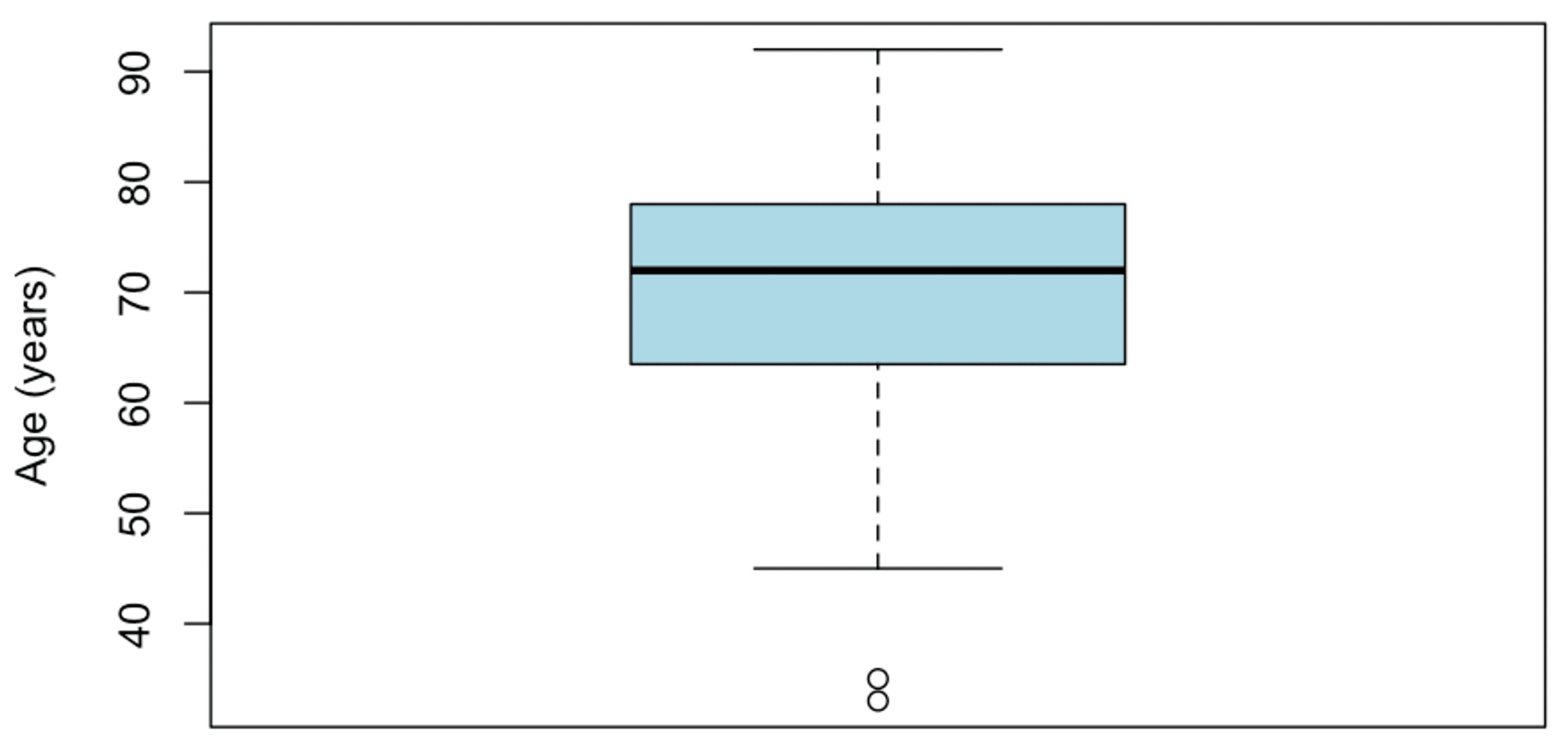
Age distribution of patients who underwent endovascular procedures for peripheral artery disease Box plot illustrating the age distribution of patients undergoing endovascular interventions for peripheral artery disease. The median age was 72 years. The distribution was non-normal, as assessed by the Shapiro-Wilk test (p = 0.014).

During 2023, 1,984 procedures were performed in the interventional radiology unit; 152 (7.7%) were PAD-related endovascular procedures performed in 115 patients. The majority of patients were male, accounting for 68 (59.13%). The median age was 72 years, and this variable had a non-normal distribution (Shapiro-Wilk test p-value = 0.014). The age distribution is presented in Figure 1.

Dyslipidemia was the most common comorbidity, documented in 93 (80.87%) patients, followed by hypertension in 84 (73.04%) and diabetes mellitus in 71 (61.74%). Other comorbidities included chronic kidney disease in 25 (21.74%), ischemic heart disease in 24 (20.87%), and heart failure in 20 (17.39%) patients. Additional comorbidities are detailed in Table 1.

**Table 1:** Baseline clinical characteristics of patients who underwent PAD-related endovascular procedures This table summarizes the demographic characteristics and comorbid conditions of patients treated with endovascular interventions for PAD. Data are presented as the number of patients and corresponding percentages. PAD, peripheral artery disease.

Variable		n (%), Total N = 115 patients
Sex		
Male		68 (59.13%)
Female		47 (40.87%)
Diabetes mellitus		71 (61.74%)
Dyslipidemia		93 (80.87%)
Hypertension		84 (73.04%)
Chronic kidney disease		25 (21.74%)
Ischemic heart disease		24 (20.87%)
Hypothyroidism		21 (18.26%)
Heart failure		20 (17.39%)
History of stroke		9 (7.83%)
Atrial fibrillation		7 (6.09%)
Cancer		7 (6.09%)
Liver disease		2 (2.61%)

Among preoperative pharmacological therapies, the most commonly documented was antihypertensive therapy, received by 75 (65.22%) patients. Statin therapy was also common; high-intensity statin use was documented in 51 (44.25%) patients. It is notable that cilostazol was prescribed in only 30 (25.86%) patients in the preoperative setting.

Treatment with anticoagulation was recorded in 44 (38.26%) patients, who were mainly on heparins. Only one patient was on a regimen with apixaban; this is of note given that atrial fibrillation was present in seven (6.09%) patients. Regarding antiplatelet therapy, 56 (48.69%) patients received preoperative management with at least one antiplatelet agent. Dual antiplatelet therapy was the most frequent antiplatelet regimen, present in 29 (25.22%) patients. Detailed pharmacological regimens are presented in Table 2.

**Table 2:** Baseline preoperative pharmacological profile of patients undergoing PAD-related endovascular procedures This table summarizes the preoperative pharmacological therapies prescribed to patients prior to endovascular intervention for PAD, including antiplatelet agents, statin therapy, antihypertensive medications, antidiabetic treatment, cilostazol use, and anticoagulation. PAD, peripheral artery disease.

Variable		n (%), Total N = 115 patients
Preoperative cilostazol use		30 (25.86%)
Antihypertensive medications		75 (65.22%)
Preoperative antidiabetic drugs		
Oral antidiabetic drugs		41 (36.65%)
Insulin		42 (36.52%)
Statin therapy		
No statin		56 (48.7%)
High-intensity		51 (44.25%)
Moderate-intensity		4 (3.48%)
Preoperative antiplatelet therapy		
No therapy		55 (47.83%)
Dual antiplatelet (clopidogrel + aspirin)		29 (25.22%)
Aspirin only		24 (20.87%)
Clopidogrel only		2 (1.74%)
Ticagrelor only		1 (0.87%)
Preoperative anticoagulation		
No therapy		68 (59.13%)
Heparins		43 (37.39%)
Apixaban		1 (0.87%)

**Figure 2:**
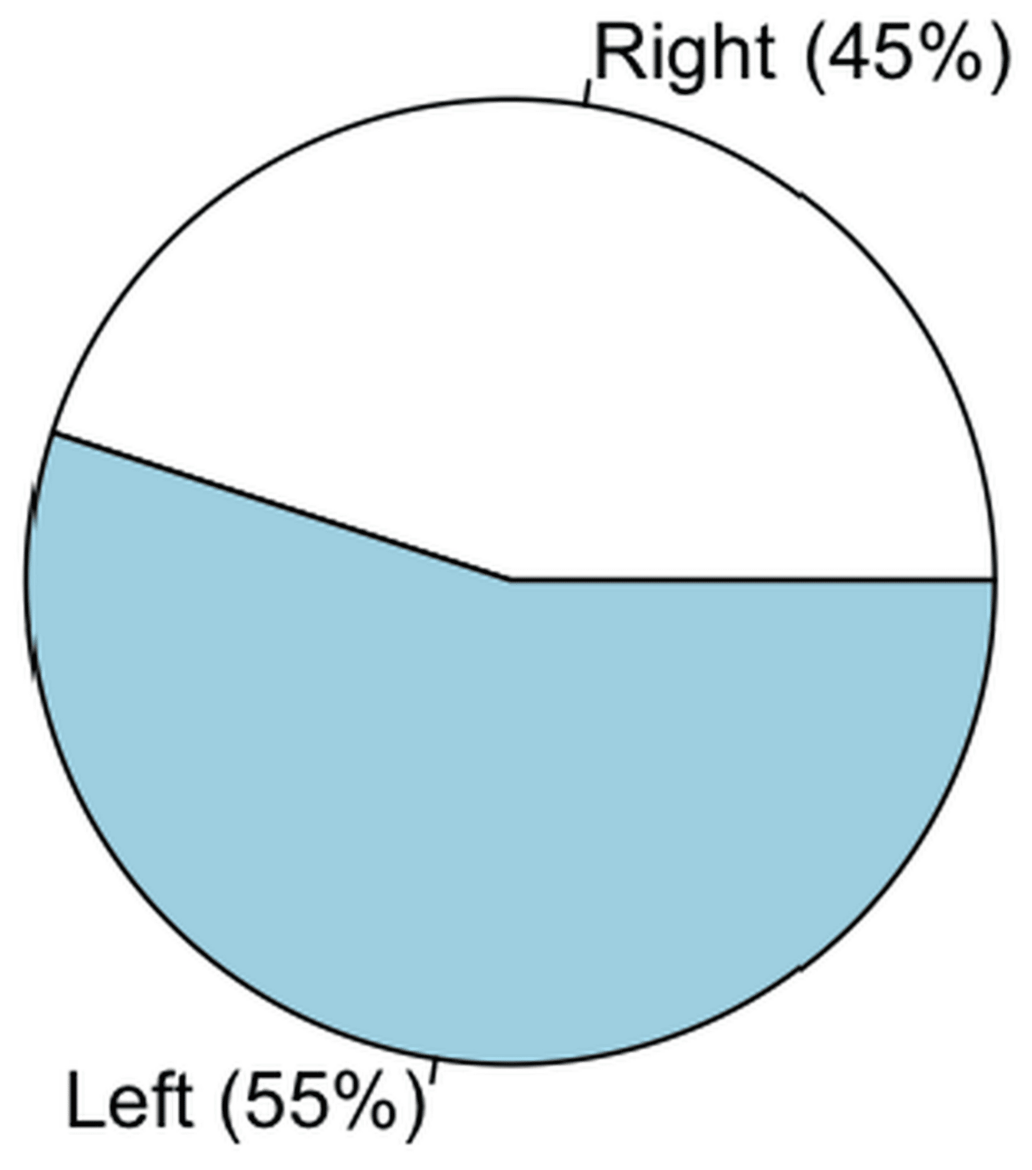
Laterality distribution of intervened limbs Pie chart showing the distribution of treated limbs among PAD-related endovascular interventions. A total of 83 (54.60%) procedures were  performed on the left limb and 69 (45.39%) on the right lower limb. PAD, peripheral artery disease.

A total of 152 procedures were included in this study. The most common indication for intervention was CLTI, with 89 (58.55%) procedures. The left lower limb was the most frequently treated, documented 83 (54.61%) times. Most procedures corresponded to an initial intervention in 112 cases (73.68%), while reinterventions accounted for 23 (15.13%). During follow-up, 16 (10.53%) limb amputations were recorded, and all were performed by the Orthopedics Department. Only one record corresponded to an angiography. Limb distribution is illustrated in Figure 2.

According to the Rutherford classification in the clinical records, 44 (28.95%) procedures were performed on patients with extensive gangrene, followed by 37 (24.34%) cases of severe claudication and rest pain in 34 (22.37%) cases. The WIfI classification showed 52 (34.21%) patients classified as grade 4, followed by 37 (24.34%) as grade 2 and 33 (21.71%) as grade 3, consistent with the findings of the Rutherford classification. Eight (5.26%) cases were classified as unsalvageable. Detailed descriptions of the obtained classifications are presented in Tables 3, 4.

**Table 3:** Periprocedural clinical characteristics and disease severity classifications of PAD-related endovascular procedures This table summarizes the laterality of treated limbs, type of intervention, clinical indication at the time of the procedure, and baseline disease severity according to the Rutherford and WIfI classification systems for all PAD-related endovascular procedures performed during the study period. PAD, peripheral artery disease; WIfI, Wound, Ischemia, and foot Infection.

Variable		n (%), Total N = 152 procedures
Intervened limb		
Left		83 (54.61%)
Right		69 (45.39%)
Event type		
First intervention		112 (73.68%)
Reintervention		23 (15.13%)
Registration of amputation date		16 (10.53%)
Diagnostic imaging procedure only		1 (0.66%)
Diagnosis at the time of intervention		
Chronic limb-threatening ischemia		89 (58.55%)
Peripheral artery disease only		41 (26.97%)
Critical ischemia		22 (14.47%)
Pre-procedure Rutherford classification		
Extensive gangrene		44 (28.95%)
Severe claudication		37 (24.34%)
At-rest pain		34 (22.37%)
Minor foot ulcers		32 (21.05%)
Moderate claudication		4 (2.63%)
Mild claudication		1 (0.66%)
WIfI classification		
Grade 4 (high risk of amputation)		52 (34.21%)
Grade 2 (low risk of amputation)		37 (24.34%)
Grade 3 (moderate risk of amputation)		33 (21.71%)
Grade 1 (very-low risk of amputation-minimal ischemia)		22 (14.47%)
Grade 5 (unsalvageable)		8 (5.26%)

**Table 4:** Angiographic classification of PAD-related endovascular procedures according to the GLASS Distribution of angiographic findings based on the GLASS classification for femoropopliteal and infrapopliteal arterial segments in 152 endovascular procedures, as well as the corresponding GLASS overall staging. Percentages are calculated based on the specified denominator for each subsection. The overall denominator is 152 procedures unless otherwise stated. Categories labeled as “No available data” correspond to missing documentation in the electronic medical records*. * GLASS, Global Limb Anatomic Staging System.

Variable		n (%), Total N = 152 procedures
GLASS classification (femoropopliteal)		
0		41 (26.97%)
3		31 (20.39%)
1		24 (15.79%)
4		23 (15.13%)
2		22 (14.47%)
No available data		11 (7.24%)
Glass classification (infrapopliteal)		
4		91 (59.87%)
3		33 (21.71%)
2		11 (7.24%)
1		3 (1.97%)
0		3 (1.97%)
No available data		11 (7.24%)
GLASS stage		
Stage 3 (1-year patency <50%)		108 (71.05%)
Stage 2 (1-year patency 50-70%)		26 (17.11%)
Stage 1 (1-year patency >70%)		5 (3.29%)
Stage 4		2 (1.32%)
No available data		11 (7.24%)

Among the performed interventions, angioplasties were the most frequently performed procedures, performed in 128 (84.21%) cases. Most interventions were performed at the infrapopliteal level, present in 66 (43.42%) cases, followed by multilevel interventions in 57 (37.50%) cases. Finally, angioplasty combined with thrombectomy was performed in nine (5.92%) cases, and thrombectomy alone was performed in only one (0.66%) case.

Regarding the endovascular devices used among angioplasties, plain balloons were the most used, documented in 132 (96.35%) cases; meanwhile, drug-coated balloons were used in five (3.64%) cases. Thirty-three cases required stent placement, where bare-metal stents were the most used, documented in 32 (96.97%) cases.

Among the 11 procedures in which complications were reported, thrombosis and acute recoil occurred in five (45.45%) cases, acute recoil in four (36.36%), and technical failure in one (9.09%) case.

**Figure 3:**
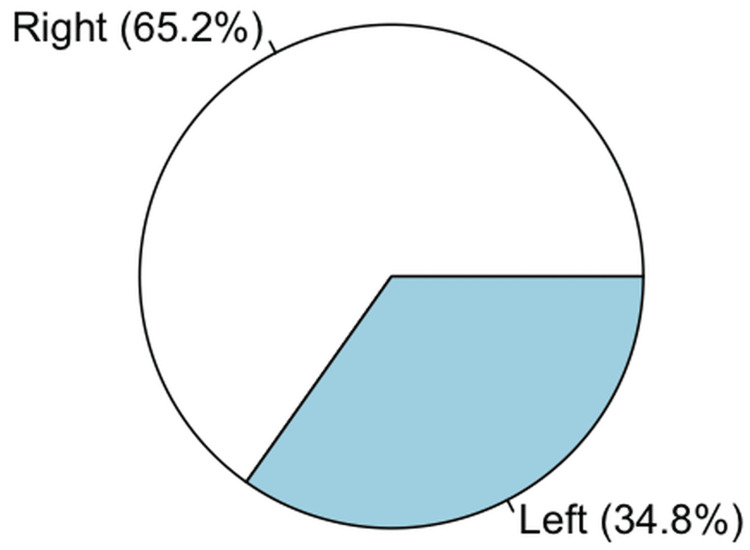
Laterality of reintervened limbs in PAD-related endovascular procedures Pie chart illustrating the laterality of limbs requiring reintervention among PAD-related endovascular procedures. Of the 23 reinterventions, 15 (65.2%) were performed on the right lower limb and 8 (34.8%) on the left lower limb. PAD, peripheral artery disease.

Among the 23 cases that required reinterventions, restenosis was the most frequent indication for reintervention, found in 12 cases (52.17%), of which three had previous stent placement. This was followed by combined thrombosis and stenosis in nine cases (39.13%), five of which had previous stent placement. Finally, isolated thrombosis was found in two cases (8.70%), none of which had previous stent placement. Limb distribution is shown in Figure 3, and additional characteristics of the performed procedures are summarized in Table 5.

**Table 5:** Procedural characteristics of PAD-related endovascular interventions This table summarizes the anatomical level of intervention, type of endovascular procedure, devices used, procedural complications, and reintervention characteristics for all PAD-related endovascular procedures performed during the study period. Percentages are calculated based on the specified denominator for each subsection. The overall denominator is 152 procedures unless otherwise stated. Categories labeled as “No available data” correspond to missing documentation in the electronic medical records. PAD, peripheral artery disease.

Variable		n (%), Total N = 152 procedures
Intervention level		
Infrapopliteal		66 (43.42%)
Multilevel		57 (37.50%)
Femoropopliteal		12 (7.89%)
Aortoiliac		5 (3.29%)
No available data		12 (7.89%)
Type of procedure performed		Total N=152
Angioplasty		128 (84.21%)
Angioplasty, thrombectomy		9 (5.92%)
Thrombectomy		1 (0.66%)
No available data		14 (9.21%)
Type of balloon used		Total N = 137
Plain balloon		132 (96.35%)
Drug-coated balloon		5 (3.64%)
Type of stent used		Total N = 33
Bare-metal stent		32 (96.97%)
Drug-eluting stent		1 (3.03%)
Referral to vascular surgery		4 (2.63%)
Complications during the procedure		Total N = 11
Thrombosis, acute recoil		5 (45.45%)
Acute recoil		4 (36.36%)
Technical failure		1 (9.09%)
Other complications		1 (9.09%)
Total number of lower-limb reinterventions		Total N = 23
1		22 (95.65%)
2		1 (4.35%)
Reason for reintervention		Total N = 23
Stenosis only		12 (52.17%)
Thrombosis and stenosis		9 (39.13%)
Thrombosis only		2 (8.70%)

Regarding postoperative pharmacological management, the most common therapy was dual antiplatelet therapy with clopidogrel and aspirin, documented in 118 (77.63%) cases. Postoperative cilostazol was prescribed in 50 (57.24%) cases, and high-intensity statin therapy in 89 (58.55%). Additional postoperative treatments found are presented in Table 6.

**Table 6:** Postoperative pharmacological management of PAD-related endovascular procedures This table summarizes postoperative pharmacological therapies prescribed following PAD-related endovascular interventions, including antiplatelet regimens, statin therapy intensity, anticoagulation, cilostazol use, and prostacyclin analog therapy. Categories labeled as “No available data” correspond to missing documentation in the electronic medical records. PAD, peripheral artery disease.

Variable		n (%), Total N = 152 procedures
Postoperative cilostazol prescription		50 (57.24%)
Postoperative statin therapy		
High-intensity statin therapy		89 (58.55%)
No statin therapy		46 (30.26%)
No available data		15 (9.87%)
Moderate-intensity statin therapy		2 (1.32%)
Postoperative antiplatelet therapy		
Dual antiplatelet therapy (clopidogrel + aspirin)		118 (77.63%)
No available data		14 (9.21%)
No antiplatelet therapy		9 (95.92%)
Aspirin only		9 (5.92%)
Dual antiplatelet therapy (ticagrelor + aspirin)		1 (0.66%)
Ticagrelor only		1 (0.66%)
Postoperative anticoagulation		
No anticoagulation therapy		93 (61.18%)
Heparins		45 (29.61%)
No available data		14 (9.21%)
Postoperative prostacyclin analogs		
No prostacyclin analogue therapy		138 (90.79%)
No available data		10 (6.58%)
Yes		4 (2.63%)

​Statistical analysis demonstrated significant associations between the WIfI classification and amputation (χ² = 61.243, p < 0.001), along with procedural complications (χ² = 87.889, p < 0.001). The Rutherford classification was significantly associated with amputation (χ² = 20.814, p < 0.001) but not with procedural complications (χ² = 6.2805, p = 0.589). The GLASS stage had a significant association with amputation (χ² = 30.078, p < 0.001) but not with procedural complications (χ² = 11.208, p = 0.511). Insulin-dependent diabetes mellitus was significantly associated with subsequent limb amputation (χ² = 6.2805, p = 0.043). Finally, the association between amputation and death was not statistically significant (χ² = 0.74361, p = 0.389). Detailed statistical analysis is presented in Table 7.

**Table 7:** Exploratory chi-square analysis of associations between clinical classification systems, procedural outcomes, limb amputation, and mortality Chi-square (χ²) tests were used to evaluate unadjusted associations between clinical classification systems (WIfI and Rutherford), angiographic classifications derived from the Global Limb Anatomic Staging System (GLASS) for femoropopliteal and infrapopliteal arterial segments, insulin-dependent diabetes mellitus, and the outcomes of procedural complications, limb amputation, and mortality. Analyses were performed at the patient level for amputation and mortality outcomes and at the procedure level for procedural complications. Statistical significance was defined as two-sided p < 0.05. WIfI, Wound, Ischemia, and Foot Infection; GLASS, Global Limb Anatomic Staging System; DM, diabetes mellitus; df, degrees of freedom.

Comparison	χ²	df	p-Value
WIfI and amputation	61.243	4	<0.001
WIfI and procedural complications	87.889	24	<0.001
Rutherford and amputation	20.814	5	<0.001
Rutherford and procedural complications	27.658	30	0.589
Femoropopliteal GLASS and amputation	4.187	4	0.381
Femoropopliteal GLASS and procedural complications	41.19	24	0.016
Infrapopliteal GLASS and amputation	7.734	4	0.102
Infrapopliteal GLASS and procedural complications	61.547	24	<0.001
Overall GLASS stage and amputation	30.078	3	<0.001
Overall GLASS stage and procedural complications	11.208	12	0.511
Insulin-dependent DM and amputation	6.281	2	0.043
Amputation and mortality	0.744	1	0.389

## Discussion

PAD is a major cause of atherosclerosis-related comorbidity; in this context, early diagnosis, timely revascularization, and risk-factor management are essential to prevent disease progression and limb amputations [6]. PAD may progress to CLTI, a condition associated with increased mortality when left untreated [7]. A higher burden of PAD has been reported in low- and middle-income countries, with a prevalence of 28.7% compared with 13.1% high-income regions [8].

In settings with limited access to specialized vascular care, the impact of PAD is especially relevant, as these regions often experience a higher prevalence of uncontrolled cardiovascular risk factors and delays in diagnosis and treatment. This underscores the importance of characterizing affected populations, such as Colombia, where healthcare resources and access to advanced vascular services may be constrained.

​​Interventional radiology has become fundamental in the management of PAD and CLTI, with early endovascular intervention playing a key role in slowing disease progression and reducing major adverse limb events [7]. During the study period, interventions related to PAD accounted for 7.7% of all the procedures performed in the interventional radiology unit, a proportion comparable to previous institutional reports from Colombia, including a prevalence of approximately 7.3% reported in Bogotá [9].

The distribution of major cardiovascular risk factors in our cohort, including diabetes mellitus, hypertension, and dyslipidemia, was consistent with national and international reports, although hypertension was more prevalent in our population [3,9,10]. These findings highlight the ongoing challenge of cardiovascular risk factor control among patients with PAD, particularly in healthcare environments with limited preventive resources.

A notable finding was the relatively high prevalence of hypothyroidism, documented in 21 (18.3%) patients. This prevalence exceeded that of prior stroke (9, 8.0%) and was comparable to heart failure (20, 17.4%). Previous studies in PAD populations and in patients with heart failure with preserved ejection fraction have reported hypothyroidism prevalence rates of approximately 5.8%, suggesting that the frequency observed in our cohort is higher than expected [11,12]. Hypothyroidism has been recognized as a cardiovascular risk factor contributing to atherosclerosis through mechanisms such as elevated low-density lipoprotein cholesterol levels, endothelial dysfunction, impaired vascular reactivity, and increased macrophage apoptosis [9-11,13,14]. Our findings suggest that hypothyroidism may represent an underrecognized comorbidity in patients with PAD and warrant further investigation. While its association with coronary artery disease is well established, its role in PAD remains insufficiently explored [15]. Similar prevalence rates of hypothyroidism have been reported in patients with end-stage kidney disease, ranging from 17% to 24% [13], which aligns closely with our findings in 25 (21.7%) patients.

The pharmacological management regimens observed in this study revealed significant gaps in secondary prevention. Preoperative cilostazol use was found in only 30 (25.86%) patients, whereas postoperative cilostazol therapy increased to 50 (57.2%) cases. This discrepancy may reflect delayed PAD diagnosis, limited outpatient follow-up, or the absence of strong guideline recommendations supporting cilostazol use during the study period. Since then, institutional practice has evolved toward standardized cilostazol prescription in accordance with emerging evidence and contemporary guidelines [16].

Among patients with diabetes mellitus, 42 (59.2%) of 71 patients were receiving insulin therapy, and among hypertensive patients, 75 (89.3%) of 84 were on antihypertensive treatment. These findings suggest suboptimal chronic disease management, which may contribute to the progression of PAD, increased procedural complexity, and adverse outcomes such as CLTI.

Regarding limb involvement, left-sided limbs and the infrapopliteal arterial segment were most frequently treated in our cohort. This contrasts with prior reports in which femoropopliteal disease predominated [17]. However, reinterventions were more commonly performed on the right limbs. Although lower-limb PAD is often considered symmetric in terms of laterality, disease distribution across arterial segments may vary according to comorbid conditions, smoking status, and overall disease burden [18-21].

Restenosis was the most frequent indication for reintervention, identified in 12 (52.2%) of 23 cases. Restenosis remains a major concern following endovascular therapy, particularly after bare-metal stent placement, with reported rates reaching up to 50% [1].

The choice of endovascular devices in this study likely reflects the local resource constraints, with plain balloon angioplasty used in most cases and limited drug-coated balloons. Despite these limitations, procedural complication rates were low, and overall outcomes were favorable. These results support the safety of endovascular therapy for PAD in healthcare systems with constrained resources.

Several clinical and anatomical classification systems were significantly associated with procedural complications and amputation, showing the importance of these classification systems as prognostic factors. These findings indicate that these staging systems primarily reflect baseline disease severity. Importantly, despite the high prevalence of advanced PAD in this cohort, complication and reintervention rates remained low, supporting endovascular management as a safe and effective strategy even in patients with complex disease.

From an interventional radiology perspective, these results emphasize the importance of interpreting adverse outcomes within the context of underlying disease burden and systemic comorbidities, rather than attributing them solely to procedural factors. The observed association between insulin-dependent diabetes mellitus and limb amputation highlights the critical role of metabolic control in determining prognosis, even after technically successful revascularization. In this context, endovascular therapy represents a valuable limb-preserving approach in regions with limited healthcare resources, where timely intervention, individualized procedural planning, and multidisciplinary care are essential. These results emphasize the need for multidisciplinary teams, including endocrinology and primary care, as optimal endovascular outcomes depend not only on technical factors but also on exhaustive systemic disease management. A multidisciplinary approach is therefore essential for improving limb salvage and long-term outcomes in this high-risk population.

This study has several limitations. Its retrospective design restricted data collection to information available in electronic medical records, resulting in missing variables, such as smoking status, in a substantial proportion of patients. The relatively small sample size limited statistical power and subgroup analyses, and the single-center design may reduce generalizability to other regions of Colombia. Despite these limitations, this study provides valuable insight into PAD management in a resource-constrained healthcare environment and contributes to the limited body of literature from Latin America.

## Conclusions

This study provides a comprehensive characterization of patients with PAD who underwent endovascular procedures in a low-resource healthcare setting. Despite the high prevalence of advanced disease stages and multiple comorbidities, complication and reintervention rates were low, highlighting the safety and favorable short-term outcomes of endovascular therapy in this patient population. These findings support the role of endovascular interventions as an effective therapeutic and preventive strategy, even among patients with complex disease profiles.

Insulin-dependent diabetes mellitus was significantly associated with limb amputation, underscoring the impact of optimal systemic disease control on clinical outcomes and the need for strengthened preventive strategies and improved metabolic management in patients with PAD. Additionally, hypothyroidism emerged as a relevant comorbidity in this population, warranting appropriate recognition and management to potentially improve outcomes. Further studies with larger cohorts and longer follow-up periods are needed to better define these associations and to optimize management strategies aimed at improving limb-salvage outcomes.

## References

[1] Gouëffic Y, Brodmann M, Deloose K, Dubosq-Lebaz M, Nordanstig J (2024). Drug-eluting devices for lower limb peripheral arterial disease. EuroIntervention.

[2] Houghton JS, Saratzis AN, Sayers RD, Haunton VJ (2024). New horizons in peripheral artery disease. Age Ageing.

[3] Castrillon-Peña EL, Poveda-Conde LC, Suaza-Vallejo MC, Vanegas-Vidal M, Barrios-Torres JC, Vargas-Plazas HI (2019). [Experience with angioplasty in lower limbs for peripheral arterial disease in a private clinic in southern Colombia]. Rev Cir.

[4] Mandaglio-Collados D, Marín F, Rivera-Caravaca JM (2023). Peripheral artery disease: Update on etiology, pathophysiology, diagnosis and treatment. Med Clin (Barc).

[5] Sarıcaoğlu MC, Aytekin B, Yiğit G, Özen A, Zafer İşcan H (2021). Endovascular balloon angioplasty for infrainguinal arterial occlusive disease: Efficacy analysis. Turk Gogus Kalp Damar Cerrahisi Derg.

[6] Aboyans V, Ricco J, Bartelink M (2018). Guía ESC 2017 sobre el diagnóstico y tratamiento de la enfermedad arterial periférica, desarrollada en colaboración con la European Society for Vascular Surgery (ESVS). Rev Esp Cardiol.

[7] Arango CG, Álvarez JCM, García JF, Correa MO (2017). [Epidemiological profile of patients with peripheral arterial disease affiliated with a health insurance entity (EPS) in Colombia in 2016]. Dissertation Thesis. [Epidemiological profile of patients with peripheral arterial disease affiliated with a health insurance entity (EPS) in Colombia in 2016]. Dissertation.

[8] Criqui MH, Matsushita K, Aboyans V (2021). Lower extremity peripheral artery disease: Contemporary epidemiology, management gaps, and future directions: A scientific statement from the American Heart Association. Circulation.

[9] Conte MS, Bradbury AW, Kolh P (2019). Global vascular guidelines on the management of chronic limb-threatening ischemia. J Vasc Surg.

[10] Urbano L, Portilla E, Muñoz W, Hofman A, Sierra-Torres CH (2018). Prevalence and risk factors associated with peripheral arterial disease in an adult population from Colombia. Arch Cardiol Mex.

[11] Duntas LH, Feldt-Rasmussen U (2025). Hypothyroidism, atherosclerosis and cardiovascular risk prevention. Nat Rev Endocrinol.

[12] da Cunha GR, Brugnarotto RJ, Halal VA, Menezes MG, Bartholomay E, Albuquerque LC, Danzmann LC (2019). Prevalence of peripheral arterial disease in patients with heart failure with preserved ejection fraction. Clinics (Sao Paulo).

[13] Ho CL, Chih HJ, Garimella PS, Matsushita K, Jansen S, Reid CM (2021). Prevalence and risk factors of peripheral artery disease in a population with chronic kidney disease in Australia: A systematic review and meta-analysis. Nephrology (Carlton).

[14] Li J, Wang Y, Luo X, Meng T, Li C, Li J, Du L (2024). Causal relationship between hypothyroidism and coronary atherosclerotic cardiovascular disease: A bidirectional two-sample Mendelian randomization. Front Cardiovasc Med.

[15] Ding N, Hua R, Guo H, Xu Y, Yuan Z, Wu Y, Li T (2025). Effect of thyroid stimulating hormone on the prognosis of coronary heart disease. Front Endocrinol (Lausanne).

[16] Gornik HL, Aronow HD, Goodney PP (2024). 2024 ACC/AHA/AACVPR/APMA/ABC/SCAI/SVM/SVN/SVS/SIR/VESS Guideline for the management of lower extremity peripheral artery disease: A report of the American College of Cardiology/American Heart Association Joint Committee on clinical practice guidelines. Circulation.

[17] Ceballos OE, Márquez JA, Messier J, Charry JP, González M (2018). [Evaluation of permeability after endovascular procedures for patients with arterial obstructions in lower limbs]. Rev Colomb Cir.

[18] Al-Sharydah AM, AlZahrani KS, Alghanimi IA, AlAnazi MM, AlHarbi RE (2023). Anatomical distribution patterns of peripheral arterial disease in the upper extremities according to patient characteristics: A retrospective cohort study. Vasc Health Risk Manag.

[19] Baretella O, Buser L, Andres C (2023). Association of sex and cardiovascular risk factors with atherosclerosis distribution pattern in lower extremity peripheral artery disease. Front Cardiovasc Med.

[20] Secchi F, Di Leo G, Delnevo A, Alì M, D'Angelo ID, Nardella VG, Sardanelli F (2020). Peripheral artery disease: How much inter-leg symmetry? A contrast-enhanced magnetic resonance angiography study. Medicine (Baltimore).

[21] Lowry D, Saeed M, Narendran P, Tiwari A (2018). A review of distribution of atherosclerosis in the lower limb arteries of patients with diabetes mellitus and peripheral vascular disease. Vasc Endovascular Surg.

